# Contributory role of viral infection in congenital tumour development

**DOI:** 10.3332/ecancer.2013.348

**Published:** 2013-09-09

**Authors:** Maryam Monajemzadeh, Soheila Sarmadi, Maryam Moeini, Mohammad Vasei, Nima Rezaei, Ata Abbasi, Reza Shahsiah, Parin Tanzifi, Maryam Eghbali

**Affiliations:** 1 Department of Pathology, Children’s Medical Center Hospital, Tehran University of Medical Sciences, Keshavarz Boulevard, Tehran 1419733151, Iran; 2 Tehran University of Medical Sciences, Infectious Disease Research Center, Tehran 1419733151, Iran; 3 Department of Pathology, Bahrami Children Hospital, Tehran University of Medical Sciences, Damavand Ave, Tehran 1641744991, Iran; 4 Research Center for Immunodeficiencies, Children’s Medical Center, Pediatrics Center of Excellence, Tehran University of Medical Sciences, Tehran 1419733151, Iran; 5 Department of Immunology, Molecular Immunology Research Center, School of Medicine, Tehran University of Medical Sciences, Tehran 1419733151, Iran; 6 Department of Pathology, Tehran University of Medical Science, Tehran 1419733151, Iran; 7 Department of Pathology, Imam Khomeini Hospital, Tehran University of Medical Sciences, Keshavarz Boulevard, Tehran 1419733151, Iran

**Keywords:** congenital tumor, virus, viral infection, PCR

## Abstract

Congenital tumours are a group of distinct infrequent disorders whose exact aetiologies have not clearly been understood so far. Viral infection seems to be one of the key factors involved in the carcinogenesis of certain tumours. This study was performed to assess whether viral DNAs are present in the congenital tumours or not. Nucleic acid from 31 congenital tumours was extracted. Detection of Epstein–Barr virus, Cytomegalovirus (CMV), adenovirus, Herpes simplex virus 1 (HSV1) and 2, Human herpes virus 6 (HHV6), and BK virus was performed using polymerase chain reaction. Viral nucleic acid was detected in eight subjects (25.8%), mostly adenovirus, CMV, and HHV6. Despite their low frequencies, a possible role could be identified for viral infections in tumour development or progression.

## Background

Tumours and tumour-like conditions in newborns occur infrequently, and they are not the same as tumours observed in older children with respect to type, incidence, clinical behaviour, and response to treatment. Although the aetiology and pathophysiology of congenital tumours are still unclear, they seem to be multifactorial; some tumours are associated with congenital anomalies, where the role of genetic factors is prominent, while environmental factors could affect the foetus during uterine life [1–4].

Viral infection seems to be one of the key factors involved in carcinogenesis, as viruses are known to be causative factors of cancer development in humans [5, 6]; some viral agents are among the aetiological factors for certain forms of cancer [7]. In addition, there is evidence for the presence of a part of viral genomes in some tumours, but obtaining a direct experimental proof seems to be difficult [8]. Different mechanisms are explained for viral carcinogenesis, including immunosuppression, oncogene activation, and tumour suppressor gene inactivation [9–11]. The association of Epstein–Barr virus (EBV), Human T-cell Lymphotropic virus (HTLV1), human herpes virus 6 (HHV6), hepatitis B virus, and hepatitis C virus with tumours has been reported [7, 12–16].

Viruses can transmit vertically from mother to infant during pregnancy or delivery [1]. Vertical transmission is common in mothers infected with human papillomavirus (HPV), while maternal HPV positivity is an important risk factor for this infection in infants [17–21]. Viral transmission in infants may have a special impact on sanitation strategies, and its association with tumourigenesis in this specific category of tumours may determine prevention and therapeutic guidelines as well.

Although there is some epidemiological evidence for virus involvement in paediatric cancers, it has not been investigated in congenital tumours to the best of our knowledge. Therefore, this study was performed to investigate the presence of some viruses in congenital tumours.

## Methods

This is a retrospective cross-sectional study on samples of a group of patients with congenital tumours during an 11-year period (January 2002 to December 2012). The pathology files of the patients admitted to the Children’s Medical Center Hospital (the Pediatrics Center of Excellence and the largest paediatric referral centre in Iran) and Bahrami Pediatric Hospital, which are both affiliated with the Tehran University of Medical Sciences, were reviewed. The inclusion criteria were the presence of a tumour at the time of birth or during the first month of life. The medical histories of all cases were obtained from the patients’ medical records. A microscopic examination of the prepared slides was performed, and proper paraffin blocks were selected.

Nucleic acid extraction was performed from two or three 5-μm sections of selected formalin-fixed paraffin-embedded tumoural tissue and also from the umbilical cords of healthy neonates as controls, using the Roche High Pure Template Preparation Kit (Roche Diagnostics, Indianapolis, Indiana, United States) according to the manufacturer’s protocol. To prevent cross-contamination, disposable blades were used for each specimen. The DNA extracts were stored at −70°C until analysis. Polymerase chain reactions (PCRs) were performed in a final volume of 20 μL; DNA content was quantified photometrically (Nanodrop, Thermoscientific 1000, United States).

Real-time PCR was performed for EBV, CMV, HSV1 and 2, HHV6, and BK virus (AmpliSens PCR kits, Bratislava, Slovak Republic) on Rotor-Gene 6000 (Corbett Research, Mortlake, Australia). For diagnosis of adenovirus, conventional PCR was performed (AmpliSens Adenovirus-Eph PCR kit, Bratislava, Slovak Republic) on Eppendorf Mastercycler (Germany) apparatus; PCR products were subjected to electrophoresis on a 2.5% agarose gel (Sigma-Aldrich, Missouri, United States) and then stained with ethidium bromide. Negative, positive, and internal controls were run in each PCR assay.

Samples from umbilical cords of normal healthy neonates were analysed as controls.

The study was approved by the Ethics Committee of Tehran University of Medical Sciences and carried out in agreement with the Helsinki Declaration.

Statistical analysis was performed using SPSS version 16.0.1 (SPSS Inc., Chicago, Illinois, United States). The statistical differences between proportions were determined by χ^2^ analysis. Numerical data were evaluated using analysis of variance, followed by Tukey’s post-hoc test. A *P* value < 0.05 was considered significant.

## Results

Thirty one cases were reviewed; 18 cases (58%) were males. The male to female ratio was 1.6:1. The mean age of the patients was 18.6 ± 9.2 days in males versus 23.5 ± 9.4 days in females; the difference was not statistically significant (range of 1–30 days, median age of 20 days). The clinical findings are summarised in Table 1.

The most common congenital tumours include teratoma (eight cases), small round cell tumour (six cases), and lymphangioma (six cases).

The great majority of investigated cases did not reveal any evidence of viral infection (23/31).

Infection was found in eight patients (25.8%). These infections were CMV in two cases, HHV6 in two cases, and adenovirus in seven cases; considering the fact that some cases had multiple viral infections, one case with multiple viral infections with adenovirus, CMV, and HHV6 and one case with adenovirus and HHV6 infections were detected in our series. The tumours containing viral infection were hamartoma (two cases), small round cell tumour (two cases), teratoma (three cases), and inflammatory myofibroblastic tumour (one case). The age range of these cases was 7 days to 1 month; four were male and four were female.

**Figure 1:**
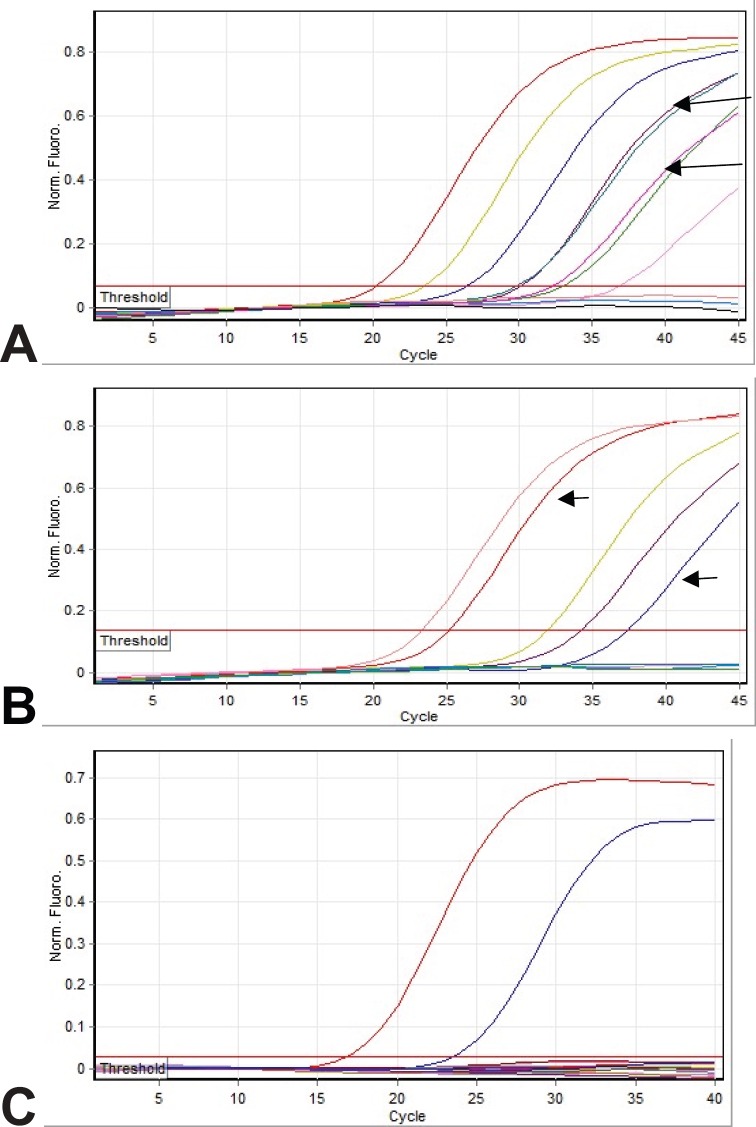
Amplification curves: (a) Real-time PCR analysis for CMV detection. Arrows show patient samples (both of them were duplicate). Other curves are positive controls. (b) Real-time PCR analysis for HHV6 detection. Arrows show patient samples. Other curves are positive controls. (c) Real-time PCR analysis for EBV detection. None of the patients’ samples show amplification. The observed curves are positive controls.

**Figure 2:**
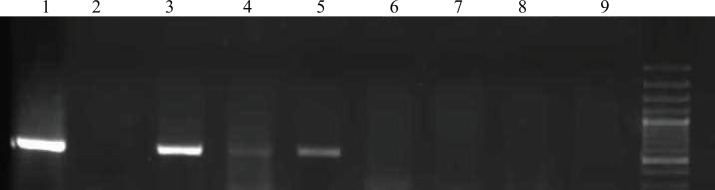
The amplification product for adenovirus was electrophoresed on agarose gel and visualised by ethidium bromide staining. Lane 1: positive control, Lanes 2 and 9: non-template controls, Lanes 3–8: patients.

Figure 1 shows the amplification curves of CMV, HHV6, and EBV. Figure 2 shows gel electrophoresis of PCR products for adenovirus detection.

None of these cases showed evidence of EBV, HSV, and BK viruses in real-time PCR examination. Also, 13 cord tissue samples obtained from normal neonates with similar weight of birth and time of delivery were gathered as controls and studied for the same viruses, and none of the infants showed any infection (*P *< 0.05).

## Discussion

Congenital neoplasms are a very rare condition, representing a spectrum of diseases different from those seen in older children suffering from cancer [1]. Furthermore, the behaviour of congenital neoplasms is different from similar tumours in older individuals; for example, some tumours with malignant histological features may reveal benign course, while histological benign neoplasms may be fatal because of their anatomical location [2].

The most common congenital tumours are haemangiomas, lymphangiomas, pigmented nevi, teratomas, neuroblastoma, leukaemia, brain tumours, and sarcomas [1]. Although the aetiology of tumours in infants may be the same as adults in general, *in utero *viral infections, history of maternal drugs and irradiation, accompanied congenital malformations, chromosomal aberrations, and also genetic predisposition could be considered as potent aetiologies of disease [1, 3].

The results of this study revealed evidence of HHV6, adenovirus, and CMV infection in congenital tumours, which are in agreement with previous findings to some extent. The most frequently isolated virus was adenovirus, which was detected in three different entities in our series, including hamartoma, small round cell tumour, and teratoma (Table 2). Adenovirus was found in multiple tumours, including sarcomas, adenocarcinomas, retinoblastomas, neuroectodermal and mammary gland tumours, and small-cell lung carcinoma [5]. It enters hosts via the mouth, nasopharynx, or conjunctiva. Subsequently, the viral DNA enters the nucleus and its early products interact with tumour suppressors such as p53 protein and retinoblastoma. Moreover, products of E3 and E4 genes are apoptosis inhibitors [5]. The carcinogenic role of adenoviruses has previously been shown in *in vitro *and *in vivo *conditions [5, 22, 23]. *In vitro *investigations showed production of adenovirus-specific proteins in transformed cells; recent *in vivo *studies revealed adenovirus serotypes in brain tumours of paediatrics [5]. Adenovirus subtypes has also been detected in human lymph node samples, involved by lymphomas and lymphocytic leukaemias [24]. 

HHV6 was detected in two of our patients. In the study by Sumiyoshi *et al*, HHV6 was found in about 50%–68% of patients with malignant lymphomas [25]. It can cause latent infection and subsequent reactivation during immunosuppression [26]. HHV was first reported as human lymphotropic virus, while further studies revealed its presence in some human tumours, especially paediatric lymphomas in association with CMV virus [26–28].

CMV was found in two of our cases presenting with inflammatory myofibroblastic tumour and hamartoma. This virus is considered as an important opportunistic infection [26]. Presence of CMV in congenital tumours has also previously been reported, suggesting its role in some neonatal sarcomas’ progression [29]. CMV infection was also detected in testicular cancer of neonates in association with EBV, suggesting the role of viral infection in testicular tumourigenesis [30].

The role of viruses in the aetiology of congenital tumours has not been well established. However, some studies suggested a possible increased risk of malignant tumours after *in utero *exposure to certain viral agents such as varicella, influenza, rubella, CMV, and human immunodeficiency virus (HIV) [1, 31]. The tumourigenicity may be related to either immune suppression or transforming activity both mediated by viruses [5]. 

Tumourigenesis is a multifactorial pathway, and viral systems support this concept that cancer development occurs by accumulation of multiple events, while the presence of viral DNA in tumour cells does not necessarily prove a causative role itself. Many pathways are introduced for viral tumourigenesis. Cell transformation caused by viral infection leading to oncogene activation or tumour suppressor gene inactivation is one of the main mechanisms for viral tumourigenesis. Oncogene production is explained for some retroviruses as their tumourigenesis mechanism, and tumour suppressor gene inactivation is introduced for some DNA viruses such as polyomavirus, papillomavirus, and adenovirus. P53 and Rb are the most important tumour suppressor genes targeted by these DNA viruses. In addition, chronic inflammation in general can lead to increased risk of cancers such as colon cancer in ulcerative colitis. Apoptosis inhibition and DNA damage are mechanisms involved in inflammation-induced malignancies. Inflammation induces proinflammatory cytokines, which in turn induce oxygen radical formation [4, 31]. The competency of neonates’ immune systems, which differ from those of adults, is another suspect in tumourigenesis [32].

In contrast to discussed findings, we found no evidence of EBV, HSV, or BK virus infections in examined tumours. There are some studies showing the presence of EBV or BK virus in some neonatal tumours, suggesting their role in tumour development or progression. EBV and CMV were detected in human lymphomas and neonatal sarcomas, respectively [26].

To the best of our knowledge, our findings provide the first evidence of the frequent occurrence of adenovirus DNA in congenital tumours. It is supposed that adenovirus leads to persistent infection after first exposure and its oncogenic properties contribute to development congenital tumours [5]. Detection of adenovirus DNA in seven tumours in comparison with other viral agents can be interpreted as false positive results and raise questions about potential contamination.

As the examined specimens were derived from two different hospitals with different times of admission, the possibility of contamination during processing and storage is minimal. On the other hand, prevention of contamination was considered when designing the project. First, in the extraction phase, a new blade and a new applicator were used for tissue sectioning and transfer, and sterile sampler tips were used for each PCR run. Second, negative control and reagent blank included in each run for the identification of contamination. Third, all PCR reactions, except for adenovirus detection, were performed in real-time format to prevent post-PCR handling and avoid contamination. Fourth, for adenovirus, for which conventional PCR was performed, post-PCR handling was done in a physically separate space. Moreover, one-way movement of staff and using dedicated equipment were the other measures for contamination prevention. Also, we performed the PCR for each positive and negative specimen on two occasions, and each time we used positive and negative controls in which the results were the same. In addition, the concentration of positive control is usually low for two reasons: first, for the identification of decreased sensitivity, for example, in longtime storage, in which case the reaction becomes negative; second, with low-concentration of positive control, the probability of contaminations decreases.

Our study had some limitations. We could not clarify whether the same virus types are present in mothers and infants to rule out potential sources of infection transmissions. One may argue that the problems with DNA amplification of archival tissues seem to be the cause of negative PCR products. Amplification of known genes as internal controls along with our target genes in all tested specimens minimised the possibility of false-negative results. Another limitation was not having the opportunity to perform sequencing to investigate the specific type or species of PCR products from the adenovirus or *in situ *hybridisation.

Finding viral genome in tumour cells does not necessarily indicate the timing or duration of infection. Peripheral blood and tissue analysis and follow-up studies should be performed to better understand the causal effect of viruses in neonatal tumour occurrence. The foetus is in danger from microbial agents that have the capacity to cross the placenta. The passage of free viral particles is one route of foetal infection. Nevertheless, these data cannot firmly conclude or exclude viral participation in congenital tumours. However, adenoviral oncoproteins can interact with the cell cycle regulation in infected cells in foetus and thereby contribute to the pathogenesis of malignant transformation. Many chronic diseases could be associated with infectious pathogens, while certain infectious pathogens are found more frequently in the patients compared with healthy controls; so pathogens associated with a disease may be suspected as a causal role in those diseases; however, association alone does not automatically prove causality. Viruses are among the few known causes of cancers, which contribute to a variety of malignancies worldwide, including the role of human papilloma virus in cervical carcinoma, human polyoma viruses in mesotheliomas and brain tumours, EBV in B-cell lymphoproliferative diseases and nasopharyngeal carcinoma, and herpes simplex virus in Kaposi’s sarcoma.

## Conclusion

The detection of some viruses in neonatal tumours could be evidence of a probable association of viral infections with tumour development or progression. The presence of adenovirus in congenital tumours but not in normal neonatal tissues suggests its possible contributory role in the development of these tumours. Meanwhile, it should be noted that there is a long way to go to discover the main and exact pathways of carcinogenesis induced by viruses and also the main carcinogenic viruses involved in this process. Further work-ups, using *in situ *hybridisation and sequence-based identification of microorganisms, can lead to confirmation and characterisations of the microbes in various tissues.

## Conflicts of interest

No conflict of interest exists.

## Figures and Tables

**Table 1.** Clinical findings of cases.

		Number	Positive PCR results
**Gender**	Male	18	4
	Female	13	4
**Tumours**	Myofibroblastic tumour	2	1
	Teratoma	8	3
	Hamartoma	4	2
	Small round cell tumour	6	2
	Granular cell tumour	2	0
	Lymphangioma	6	0
	Hepatoblastoma	2	0
	Choroid plexus papilloma	1	0

PCR, polymerase chain reaction.

**Table 2.** Viruses in various tumoural tissues

	Adenovirus	EBV	CMV	BK virus	HHV6	HSV
**Teratoma**	3	0	0	0	0	0
**Small round cell tumour**	2	0	0	0	0	0
**Hamartoma**	2	0	1	0	2	0
**Myofibroblastic tumour**	0	0	1	0	0	0

EBV: Epstein–Barr virus; HHV: human herpes virus; CMV: cytomegalovirus;

HSV: herpes simplex virus.
